# Chemotherapy induced neutropenia at 1-month mark is a predictor of overall survival in patients receiving TAS-102 for refractory metastatic colorectal cancer: a cohort study

**DOI:** 10.1186/s12885-016-2491-y

**Published:** 2016-07-13

**Authors:** Pashtoon M. Kasi, Daisuke Kotani, Michael Cecchini, Kohei Shitara, Atsushi Ohtsu, Ramesh K. Ramanathan, Howard S. Hochster, Axel Grothey, Takayuki Yoshino

**Affiliations:** Division of Medical Oncology, Mayo Clinic, 200 First St SW, Rochester, 55905 MN USA; Department of Gastrointestinal Oncology, National Cancer Center Hospital East, Kashiwa, Chiba Japan; Division of Medical Oncology, Yale Cancer Center, New Haven, CT USA; Division of Medical Oncology, Mayo Clinic, Scottsdale, Arizona USA

**Keywords:** TAS-102, Colorectal Cancer, Chemotherapy induced neutropenia, Hematological toxicity, Biomarker, Predictive biomarker, Prognostic marker, Pharmacogenomics

## Abstract

**Background:**

TAS-102 (trifluridine and tipiracil hydrochloride; a novel combination oral nucleoside anti-tumor agent) has recently received regulatory approval for patients with refractory metastatic colorectal cancer (mCRC). Internal review of data at a single-institution showed a trend towards better overall survival (OS) for patients who experienced chemotherapy-induced neutropenia at 1-month (CIN-1-month). To explore this finding further, a cohort study was designed based on outcome data from three centers in United States and one from Japan.

**Methods:**

CIN-1-month after starting TAS-102 was defined by the Common Terminology Criteria for Adverse Events (CTCAE), version 4.03 as a neutrophil count decrease of ≥ grade 2 (absolute neutrophil count < 1500/mm^3^). Patients had confirmed mCRC that was refractory to standard therapies. Patient demographics and clinical characteristics were compared between patients with CIN-1-month (*CIN-1-month positive*) versus those who did not have CIN-1-month (*CIN-1-month negative*); with the median progression-free survival (PFS) and OS were calculated using the Kaplan-Meier method, and differences evaluated using the Log-rank test.

**Results:**

Our cohort study had a total of 149 patients with data regarding their neutrophil assessment at 1-month mark. Patients who developed ≥ grade 2 CIN-1-month had a both longer PFS (median 3.0 months versus 2.4 months; Log-rank *P*-value = 0.01), as well as OS (14.0 versus 5.6 months; Log-rank *P*-value < 0.0001). Only CIN-1-month (adjusted HR: 0.21 (95 % CI: 0.11–0.38) and higher baseline CEA levels (adjusted HR: 2.00 (95 % CI: 1.22–3.35) were noted to be independent predictors of OS. Furthermore, the CIN-1-month was noted to be a statistically significantly predictor of OS over a wide range of cutoffs.

**Conclusions:**

Our observations are novel and hypothesis generating. Neutropenia after starting TAS-102 was associated with better prognosis in patients with refractory mCRC. It can be postulated that the dosage of TAS-102 potentially may need to be increased to achieve better outcomes in patients not experiencing any neutropenia. Further pharmacologic investigations should help elucidate these issues.

## Background

TAS-102 (trifluridine and tipiracil hydrochloride; a novel combination oral nucleoside anti-tumor agent) was first approved in Japan in March 2014 and received US Food and Drug Administration (FDA) approval in September 2015 after an international phase-III clinical trial in patients with refractory metastatic colon cancer demonstrated a benefit in overall survival for TAS-102 compared with placebo [1]. Prior to the FDA approval, patients had access to an expanded access program (EAP) of TAS-102 at various institutions within United States. Internal review of outcome data at Mayo Clinic in patients who were treated through the EAP showed a trend towards longer progression free survival (PFS) and overall survival (OS) for patients who were noted to have neutropenia after one cycle (4 weeks) of therapy. To explore this finding further, we validated these findings with outcomes data on additional patients who had received TAS-102 at the Yale Cancer Center, United States, as well as at the National Cancer Center Hospital East, Japan, where the drug had been approved in 2014.

**Fig. 1:**
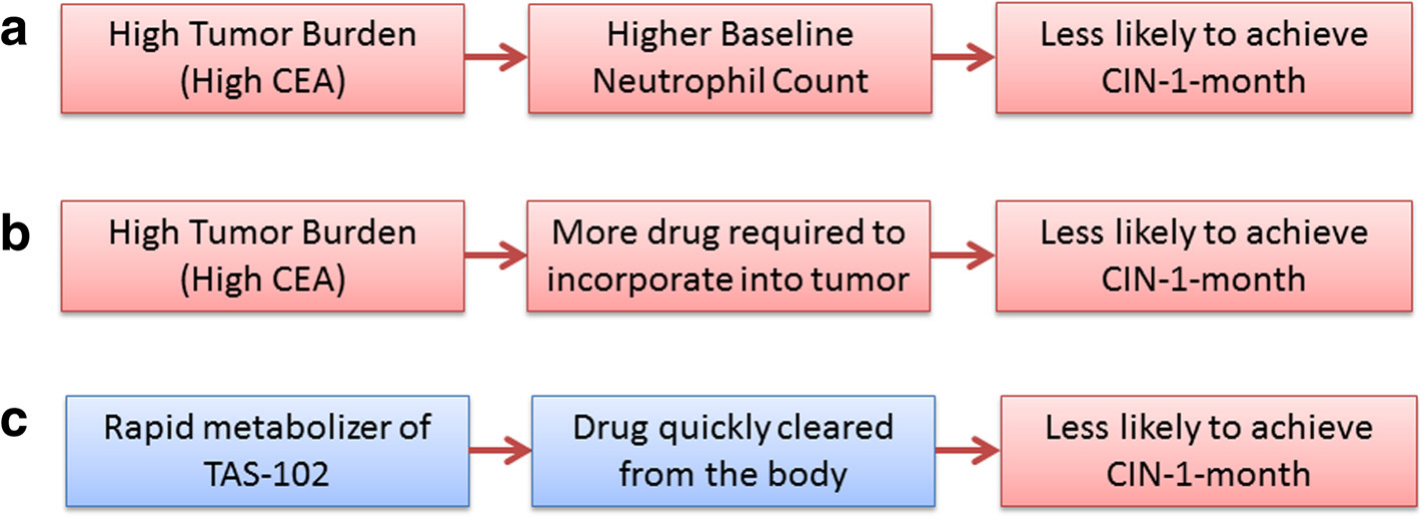
Postulated mechanisms between association of chemotherapy-induced neutropenia at 1-month mark (CIN-1-month) and overall survival: (**a**) Firstly, one may postulate that patients with a high tumor burden could have a high baseline neutrophil count; making it less likely to experience CIN-1-month; (**b**) Secondly, since the drug incorporates into the tumor, for patients with a high tumor burden, it is possible that that the standard dosage of the drug may not be enough to exert myelotoxicity; and **c**) Finally, individuals experiencing different degrees of neutropenia may have different pharmacokinetics of TAS-102 and its metabolites

Chemotherapy induced neutropenia (CIN) at 1-month mark [CIN-1-month] after starting TAS-102 was defined by the Common Terminology Criteria for Adverse Events (CTCAE), version 4.03 as a neutrophil count decrease of ≥ grade 2 (absolute neutrophil count < 1500/mm^3^). Our hypothesis was that the hematological toxicity (CIN-1-month) was a predictive marker of outcomes in patients with refractory metastatic colorectal cancer through several potential mechanisms (Fig. 1). In fact, in a recently published preclinical model, trifluridine (TFT; which is the anti-tumor component of TAS-102) incorporated itself in the DNA of the colorectal tumor as well as the DNA of the white blood cell in a dose dependent manner [2]. The highest tolerable TFT concentration was the one that provided the highest anti-tumor activity, with hematological toxicity as a potential surrogate marker for the effectiveness of the drug [2]. If our hypothesis is valid, patients who do not have chemotherapy induced neutropenia should have a higher risk of death. The results would provide further rationale to these observations. It will further elucidate the mechanism of action of the drug responsible for its anti-tumor activity. Furthermore, the clinical observation of CIN-1-month would have multiple potential therapeutic implications [3].

## Methods

### Patients

Patients enrolled in the EAP cohort were age 18 years or older with confirmed metastatic adenocarcinoma of the colon or rectum, and an Eastern Cooperative Oncology Group (ECOG) performance status of 0 or 1. Patients needed to have previously progressed during or within 3 months following the last administration of approved standard therapies which must have included fluoropyrimidine, oxaliplatin, irinotecan, bevacizumab or aflibercept and cetuximab or panitumumab if *RAS* wild-type. Patients who had withdrawn from standard treatment due to unacceptable toxicity warranting discontinuation of treatment and precluding retreatment with the same agent prior to progression of disease were also allowed to enter the EAP. The detail of the eligibility criteria for the EAP that was open at 33 different sites within United States is available at http://clinicaltrials.gov (identifier: NCT02286492) [4]. For the purpose of this study, our EAP cohort comprised of patients from three cancer centers in United States (Mayo Clinic Rochester, Mayo Clinic Arizona and Yale Cancer Center).

### Expanded Access Program (EAP) cohort from United States

Our ‘EAP cohort’ comprised of patients who were enrolled through the expanded access program (EAP) of TAS-102 at the Mayo Clinic (Rochester and Arizona sites, United States) and the Yale Cancer Center, New Haven, Connecticut, United States between March 04, 2015 and September 30, 2015. Institutional Review Board (IRB) approvals were obtained from both institutions prior to the initiation of the EAP, alongside approval from Taiho Pharmaceutical Co. LTD.

### Validation cohort from Japan

Our ‘validation cohort’ included patients with histologically confirmed colorectal adenocarcinoma who were treated with TAS-102 in Japan from May 01, 2014 to September 30, 2015 [5]. The retrospective data from the validation cohort was collected under an IRB waiver in accordance with the Japanese Ethical Guidelines for Epidemiological Research.

### Study design

To answer the question if CIN-1-month affects outcomes in patients with refractory metastatic colorectal cancer, a cohort study was performed. All patients were treated with TAS-102 at a dose of 35 mg/m^2^ administered orally twice daily for 5 days a week with 2 days’ rest for 14 days, followed by a 14-day rest (1 treatment cycle). Patients with chemotherapy induced neutropenia (CIN) at 1-month mark [CIN-1-month] after starting TAS-102 as defined by the Common Terminology Criteria for Adverse Events (CTCAE), version 4.03 as a neutrophil count decrease of ≥ grade 2 (absolute neutrophil count < 1500/mm^3^) were defined as the *CIN-1-month positive group*. Patients without CIN-1-month were the reference group (*CIN-1-month negative group*). Patients were subsequently assessed for the different outcomes as described.

### Endpoints and assessments

The medical records of patients were retrospectively reviewed by investigators at the four institutions and data abstracted for the purposes of this study to gather data regarding PFS, OS and outcome of their first imaging computed tomography (CT) scans. PFS was defined as the interval from the start of TAS-102 treatment to either disease progression or death. OS was defined as the interval from the start of TAS-102 treatment to death. For PFS or OS, the patients were censored at their last follow-up visit if they were free of disease progression or death, respectively [5]. The median PFS and OS were calculated using the Kaplan-Meier method, and differences between patients with and without CIN-1-month were evaluated using the Log-rank test. Data from both cohorts were combined for further reporting and analysis. Additionally, separate subset analyses were also conducted for the endpoints described. Patients with less than 4 weeks (28 days) of follow up were excluded from the final analysis. Statistical analysis was performed using JMP®10.0 (2012 SAS Institute Inc.). Laboratory data regarding absolute neutrophil counts and carcinoembryonic antigen (CEA) level were also collected if available at baseline and on day 1 of initiation of different cycle visits. Based on the median, patients were divided into older adults (age >65) and young adults (age ≤65), high CEA levels (CEA >55 ng/ml) and low CEA levels (≤55 ng/ml), and high baseline neutrophil count (> 4300/mm^3^) and low baseline neutrophil count (≤ 4300/mm^3^). Sensitivity analyses were also conducted based on different cutoffs of neutrophil count at 1-month mark to assess the relationship between hematologic toxicity and overall survival.

## Results

### Patients

The EAP cohort had a total of 83 patients (49 patients from the two Mayo Clinic sites and 34 from the Yale Cancer Center). The validation cohort from Japan had 92 patients. Thus, the study included a total of 175 individuals. Excluding patients with less than 4 weeks (28 days) of follow up (18 patients; 10 %), our final cohort had a total of 157 patients. Data on neutrophil counts at the 4-week mark were available in 149 patients, 69 (46 %) of which experienced neutropenia (*CIN-1-month positive*) and 80 (54 %) who did not (*CIN-1-month negative*).

### Comparison between EAP and validation cohorts

We compared baseline characteristics and outcome data between the EAP cohort from United States and the validation cohort from Japan to see if there were any differences that might be explained by patients from different origins. Patient in the EAP cohort were noted to be younger (median age 61 years versus 65 years; *P*-value = 0.08). No statistically significant differences were noted between the two cohorts in the demographic characteristics or outcomes (data not shown). Therefore, further analyses reported in the paper are on the 2 cohorts combined.

### Efficacy

A total of 144 patients had their first staging imaging scans (~ after 2 cycles of therapy) available for review. At first evaluation, 84 (58 %) patients had progressive disease (PD), 55 (38 %) patients had stable disease (SD) and 5 (4 %) patients had a partial response (PR) to TAS-102. The median overall survival (OS) and progression-free survival (PFS) was 8.9 months and 2.6 months; comparable to the 7.1 month and 2.0 months in the original phase-III study [1]. Detailed data on safety and outcomes of 55 of the 92 patients were recently published by the authors from Japan [5].

### Chemotherapy Induced Neutropenia (CIN) - at 1-month mark

**Fig. 2:**
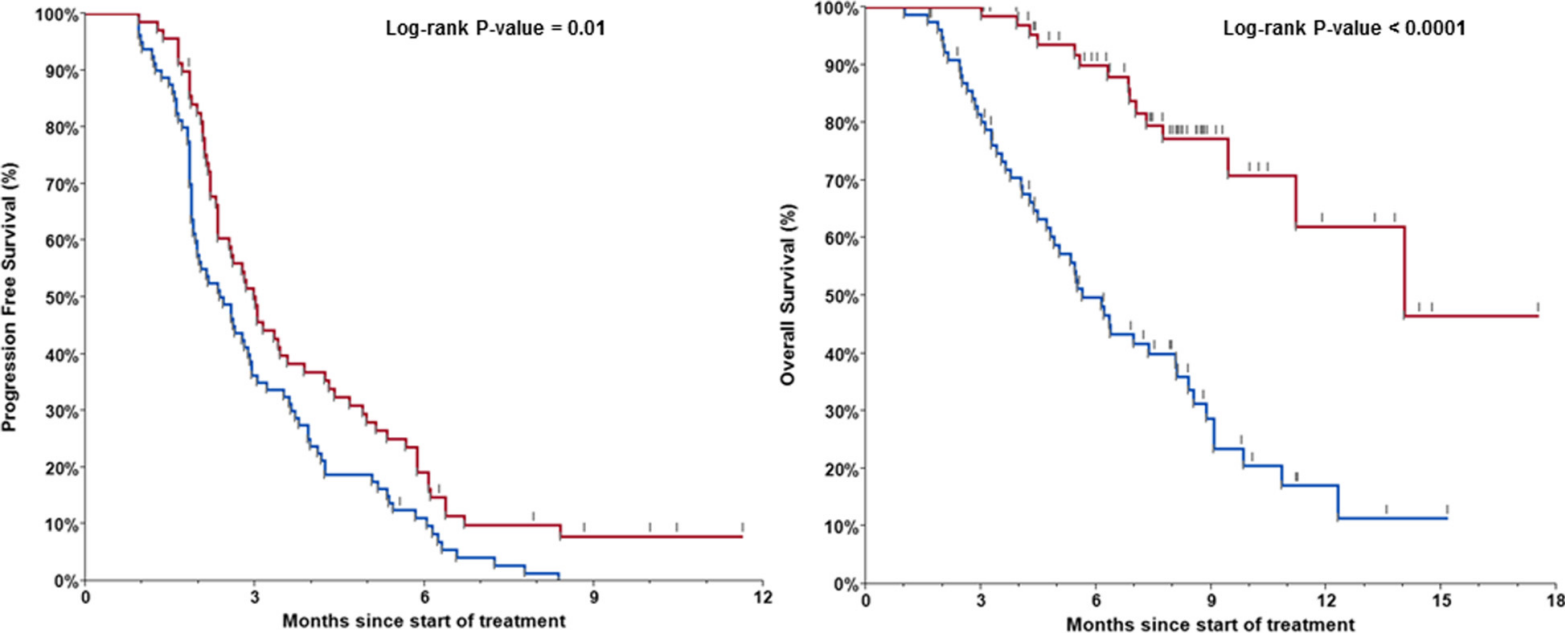
Kaplan-Meier curves for progression-free survival (PFS; median 3.0 months versus 2.4 months; *Log-rank P-value = 0.01*) as well as overall survival (OS; median 14.0 months versus 5.6 months; *Log-rank P-value < 0.0001*) for patients who achieved chemotherapy induced neutropenia (CIN) ≥ grade 2 CTCAE (*red line* – 69 patients (46.3 %) – *CIN-1-month positive*) as compared to those who did *NOT* have ≥ grade 2 CIN at the 1-month mark (*blue line* – 80 patients (53.7 %) – *CIN-1-month negative*) after starting treatment with TAS-102

Table 1 summarizes the characteristics of patients with chemotherapy-induced neutropenia at 1 month (*CIN-1-month positive*) and those who did not achieve chemotherapy-induced neutropenia at 1 month (*CIN-1-month negative*). A total of 69 (46.3 %) patients developed ≥ grade 2 CIN at 1 month-mark. Patients who developed ≥ grade 2 CIN-1-month had a both longer progression-free survival (median 3.0 months versus 2.4 months; Log-rank *P*-value = 0.0096; Fig. 2) as well as overall survival (14.0 versus 5.6 months; Log-rank *P*-value < 0.0001; Fig. 2). Additionally, the number of *CIN-1-month positive* patients achieved disease control was 32 (49.2 %) as compared to 28 (37.8 %) in the *CIN-1-month negative* group (*P*-value = 0.18). There were no significant differences in the sex, older adults, primary site (colon versus rectum) and *RAS*-mutational status between the two cohorts.

**Table 1:** Comparison of patients with metastatic colorectal cancer who achieved chemotherapy induced neutropenia (CIN) ≥ grade 2 CTCAE (*EXPOSED – CIN-1-Month positive*) as compared to those who did *NOT* have ≥ grade 2 CIN at the 1-month mark – (*Referent – CIN-1-Month – ve*) after starting treatment with TAS-102

	Variable	Patients with CIN-1-month N (%) (CIN-1 month positive)	Patients without CIN-1-month N (%) (CIN-1 month - ve)	*P*-value
1	Number	69 (46.3 %)	80 (53.7 %)	
2	Females	29 (42.0 %)	42 (52.5 %)	0.20
3	Older Adults (Age > 65 years)	39 (56.5 %)	39 (48.8 %)	0.34
4	EAP (versus validation cohort)	33 (47.8 %)	37 (46.3 %)	0.85
5	Primary site Colon (vs. Rectal)	38 (55.1 %)	50 (62.5 %)	0.36
6	*RAS*-wild type	32 (46.4 %)	41 (51.3 %)	0.56
7	High Baseline CEA (> 55 ng/ml)	24 (34.7 %)	43 (53.8 %)	0.02*
8	Higher Baseline Neutrophil Count (> 4300/mm^3^)	19 (27.5 %)	55 (68.8 %)	<0.0001**
9	Overall disease control rate (DCR)	32 (49.2 %)	28 (37.8 %)	0.18
	-Partial Response (PR)	-4 (6.1 %)	-1 (1.3 %)	
	-Stable Disease (SD)	-28 (43.1 %)	-27 (36.5 %)	
	-Progressive Disease (PD)	-33 (50.8 %)	-46 (62.2 %)	
10	Progression Free Survival (PFS) in months (95 % CI^2^)	3.0 (2.3–3.6)	2.4 (1.9–2.9)	0.0096*
11	Overall Survival (OS) in months (95 % CI^2^)	14.0 (11.2-NR^1^)	5.6 (4.7–8.1)	<0.0001**

^1^ *NR* not reached, ^2^ *CI* confidence interval

*p-value < 0.05

**p-value < 0.001

Hazard ratios (HR) for overall survival alongside 95 % confidence intervals (CI) were estimated through the Cox proportional hazards model (Table 2). Separate analyses for different stratum are shown. Only CIN-1-month (adjusted HR: 0.21; 95 % CI: 0.11–0.38: *P*-value < 0.0001) and ‘Higher Baseline CEA’ (adjusted HR: 2.00; 95 % CI: 1.22–3.35: *P*-value 0.0062) were noted to be independent predictors of overall survival. All *P*-values for interaction were higher than 0.20. Overall survival was significantly different between the *CIN-1-month positive* and *CIN-1-month negative* patients in both the ‘Higher Baseline CEA’ (9.4 months versus 4.5 months; *P*-value 0.0006) and ‘Lower Baseline CEA’ (median not reached versus 8.0 months; *P*-value 0.0003) groups of colorectal cancer patients. Furthermore, the CIN-1-month was noted to be a statistically significantly predictor of overall survival over a wide range of cutoffs (Table 2).

**Table 2:** Association between CIN-1-month and overall survival in patients with metastatic colorectal cancer receiving TAS-102

Sample or stratum	HR (95 % CI)^a^	*P*-value
Overall	0.22 (0.12–0.38)	< 0.0001
Strata		
Men	0.15 (0.06–0.33)	< 0.0001
Women	0.31 (0.13–0.67)	0.0023
Rectal	0.36 (0.15–0.78)	0.0092
Colon	0.15 (0.06–0.34)	< 0.0001
RAS-mutant	0.32 (0.15–0.64)	0.001
RAS-wild type	0.14 (0.05–0.35)	< 0.0001
Older adults (age > 65 years)	0.19 (0.09–0.40)	< 0.0001
Younger adults (age ≤ 65 years)	0.21 (0.07–0.52)	0.0003
Higher Baseline CEA (> 55 ng/ml)	0.26 (0.11–0.56)	0.0004
Lower Baseline CEA (≤ 55 ng/ml)	0.18 (0.07–0.42)	< 0.0001
Higher Baseline Neutrophil Count (> 4300/mm^3^)	0.25 (0.08–0.57)	0.0005
Lower Baseline Neutrophil Count (≤ 4300/mm^3^)	0.27 (0.11–0.65)	0.0035
Absolute Neutrophil cutoff at 1-month		
CIN-1-month (< 1000/mm^3^)	0.34 (0.16–0.66)	0.0008
CIN-1-month (< 1500/mm^3^)	0.22 (0.12–0.38)	< 0.0001
CIN-1-month (< 2000/mm^3^)	0.28 (0.17–0.47)	< 0.0001
CIN-1-month (< 2500/mm^3^)	0.25 (0.16–0.42)	< 0.0001
CIN-1-month (< 3000/mm^3^)	0.26 (0.16–0.43)	< 0.0001

^a^HR for overall survival was calculated through Cox proportional hazards models

## Discussion

Our study suggests that chemotherapy induced neutropenia at 1-month mark (CIN-1-month) after starting TAS-102 appears to be a prognostic and/or predictive biomarker of both PFS and OS in patients with refractory metastatic colorectal cancer. Individuals who developed CIN-1-month had a significant improved survival (14.0 versus 5.6 months; *P*-value < 0.0001).

In both the previously conducted phase-II and the recently published phase-III study, neutropenia was the most common adverse event in patients who received TAS-102 [1, 6]. This required at least one dose reduction and/or a treatment interruption in up to a third of patients [7–9]. Similar safety and efficacy was noted in subsequent studies [5].

Although the mechanism underlying the association of CIN-1-month and OS in patients with refractory metastatic colorectal cancer is not entirely clear, three hypotheses can be postulated (Fig. 1). Firstly, one may postulate that patients with a high tumor burden could have a high baseline neutrophil count; making it less likely to experience CIN-1-month. Secondly, since the drug incorporates into the tumor, for patients with a high tumor burden, it is possible that that the standard dosage of the drug may not be enough to exert myelotoxicity. Finally, individuals experiencing different degrees of neutropenia may have different pharmacokinetics of TAS-102 and its metabolites. Our analyses, however, showed that the CIN-1-month was still statistically significantly associated with OS after controlling for tumor burden and other potential confounders.

Based on these observations, one can postulate that the dosage of TAS-102 may need to be increased in patients not experiencing any neutropenia to improve outcomes. Conversely, one may consider increasing the interval of chemotherapy instead of decreasing the TAS-102 dose in the subset of patients having significant decline in their absolute neutrophil counts without any clinical complications [10]. Prophylactic antibiotics and the use of growth factor support with a different dosing schedule may be other considerations, especially when considering combining this novel agent with other chemotherapy regimens for potential future clinical trials [11].

Of note, correlation between chemotherapy induced toxicities and favorable outcomes have been described previously in a number of different settings [12, 13]. Whether this is purely related to pharmacokinetics of the drug or other proposed mechanisms as outlined above remains to be determined.

Our study, however, has several limitations. First, our sample size was relatively small for some of the stratified analyses as shown by wide confidence intervals. Follow up for some of the patients in the EAP cohort is still relatively short. The majority of the patients, however, had already progressed on the study drug given the highly refractory nature of the population under study.

Our observation is hypothesis generating and has a number of strengths. First, it was based on a prospectively enrolled EAP cohort as part of the expanded access clinical trial with similar cohort of patients with refractory colorectal cancer. Second, we were able to corroborate the observations in an independent cohort of patients from a different center as well as a different country. Third, analyses were stratified for several known prognostic factors and potential confounding effects were explored. Validation of our findings in an independent population cohort is the strength of this analysis and provides a readily available potentially predictive as well as prognostic biomarker (CIN-1-month) for patients with metastatic colorectal cancer.

## Conclusions

Neutropenia after starting TAS-102 was associated with better prognosis in patients with refractory mCRC. Our findings are clinically relevant and have led to re-analyses of both the initial randomized phase-II (Study J003-10040030) and phase-III (RECOURSE trial) studies of TAS-102 versus placebo, and similar results were seen. These findings are important since it can be postulated that the dosage of TAS-102 potentially may need to be increased to achieve better outcomes in patients not experiencing any neutropenia. Further pharmacologic investigations should help elucidate these issues and help validate the potential utility of CIN-1-month as a prognostic and/or predictive biomarker of TAS-102 for patients with refractory mCRC.

## Abbreviations

CEA, carcinoembryonic antigen; CIN-1-month, chemotherapy-induced neutropenia at 1-month; CTCAE, common terminology criteria for adverse events; EAP, expanded access program; mCRC, metastatic colorectal cancer; OS, overall survival; PFS, progression-free survival; TAS-102, (trifluridine and tipiracil hydrochloride; a novel combination oral nucleoside anti-tumour agent)
